# Supplementary material for: Physiologically Based Pharmacokinetic (PBPK) Modeling for Predicting Brain Levels of Drug in Rat
Source: Pharmaceutics. 2021 Sep 3;13(9):1402. doi: 10.3390/pharmaceutics13091402 (PMC8471455; doi:10.3390/pharmaceutics13091402)
Supplement: Supplementary file 1 [file pharmaceutics-13-01402-s001.zip › pharmaceutics-1305565-supplementary.pdf]

pharmaceutics

MDPI

Supplementary Materials: Physiologically Based Pharmacokinetic (PBPK) Modeling for Predicting Brain Levels of Drug in Rat

Bárbara Sánchez-Dengra, Isabel González-Álvarez, Marival Bermejo * and Marta González-Álvarez

Table S1. Chromatographic conditions.

Drug	C (µM)	Wavelength	Mobile Phase	Retention Time (min)	r^2	LLQ (µM)	Accuracy	Precision	Ref.
Amitriptyline	250	240 nm	40% Acid water<br>60% Acetonitrile	1.020	0.996	8.20	6.1	3.2	[1,2]
Caffeine	2.14	273 nm	35% Methanol<br>65% Acid water	1.200	0.999	0.05	3.1	4.3	[3]
Carbamazepine	18	280 nm	65% Acid water<br>35% Acetonitrile	1.926	0.994	0.76	3.9	3.6	[2]
Fleroxacin	1.39	285 nm	70% Acid water<br>30% Acetonitrile	1.348	0.997	0.05	6.0	5.2	[2]
Pefloxacin	8.91	285 nm	65% Acid water<br>35% Acetonitrile	0.721	0.998	0.61	3.9	3.7	[2]
Zolpidem	158	231 nm	60% Water<br>20% Methanol<br>20% Acetonitrile	4.624	0.997	4.30	6.3	4.8	[2]

Acid water had 0.05% (*v*/*v*) trifluoroacetic acid.

Table S2. Molecular and physicochemical properties and transporters information for the studied drugs.[4,5].

Drug	MW (g/mol)	Solubility logS (pH 7)	logP	Strongest Acidic pKa	Strongest Basic pKa	Charge (pH 7.4)	Transporters (substrates)
Amitriptyline	277.411	−1.63	4.81		9.76	+	ABCB1 (Pgp)
Caffeine	194.194	−0.44	−0.55		−1.16	0	
Carbamazepine	236.274	−3.79	2.77	15.96		0	ABCC2 RALBP1
Fleroxacin	369.344	−1.33	0.98	5.32	5.99	−	
Pefloxacin	333.363	−1.21	0.75	5.5	6.44	−	ABCB1 (Pgp)
Zolpidem	307.397	−4.27	3.02		5.39	0	

MW = molecular weight

References

1. Mangas-Sanjuan, V.; González-Álvarez, I.; González-Álvarez, M.; Casabó, V.G.; Bermejo, M. Innovative in vitro method to predict rate and extent of drug delivery to the brain across the blood-brain barrier. *Mol. Pharm.* **2013**, *10*, 3822–3831, doi:10.1021/mp400294x.
2. Sánchez-Dengra, B.; González-Álvarez, I.; Sousa, F.; Bermejo, M.; González-Álvarez, M.; Sarmento, B. In vitro model for predicting the access and distribution of drugs in the brain using hCMEC/D3 cells. *Eur. J. Pharm. Biopharm.* **2021**, *163*, 120–126, doi:10.1016/j.ejpb.2021.04.002.
3. del Moral-Sanchez, J.; Ruiz-Picazo, A.; Gonzalez-Alvarez, M.; Navarro, A.; Gonzalez-Alvarez, I.; Bermejo, M. Impact on intestinal permeability of pediatric hyperosmolar formulations after dilution: Studies with rat perfusion method. *Int. J. Pharm.* **2019**, *557*, 154–161, doi:10.1016/J.IJPHARM.2018.12.047.
4. Chemicalize Chemicalize - Instant Cheminformatics Solutions Available online: https://chemicalize.com/welcome (accessed on 3 May 2020).
5. DrugBank DrugBank Available online: https://www.drugbank.ca/ (accessed on 14 May 2020).
